# A supervised exercise intervention during cancer treatment for adolescents and young adults—FiGHTING F!T: study protocol of a randomised controlled trial

**DOI:** 10.1186/s13063-021-05616-8

**Published:** 2021-10-03

**Authors:** Claire Munsie, Jay Ebert, Joanne Collins, Megan Plaster, David Joske, Timothy Ackland

**Affiliations:** 1 School of Human Sciences (Exercise and Sport Science), University of Western Australia, Perth, Western Australia Australia; 2 Western Australian Youth Cancer Service, Perth, Western Australia Australia; 3 Sir Charles Gairdner Hospital, Perth, Western Australia Australia; 4 School of Medicine, University of Western Australia, Western Australia Perth, Australia

**Keywords:** Exercise, Treatment-related toxicity, Rehabilitation, Physical activity

## Abstract

**Background:**

High-quality evidence supports the integration of exercise to mitigate treatment-related side effects in a wide range of paediatric and adult cancer cohorts. However, the implementation of exercise in adolescent and young adult (AYA) cancer patients is yet to be explored in depth. FiGHTINGF!T is a randomised controlled cross over trial designed to determine if a supervised, structured, and progressive exercise programme can reduce the decline in physical fitness (V0_2peak_) associated with cancer treatment in AYAs from diagnosis.

**Methods/design:**

A total of 40 AYAs recently diagnosed and due to commence systemic treatment (± 2 weeks) for a primary haematological malignancy or solid tumour will be recruited and randomised to either an immediate exercise intervention or usual care (delayed exercise) for 10 weeks. This randomised controlled crossover trial will see both groups engage in a supervised exercise intervention from either diagnosis (baseline assessment) for 10 weeks (0–10 weeks) or following an interim assessment to 20 weeks (10–20 weeks). The bi-weekly tailored exercise programme will combine aerobic and resistance exercises and be supervised by an Accredited Exercise Physiologist. Participants will complete a range of assessments at 0, 10, and 20 weeks including cardiopulmonary exercise tests, 1 repetition maximum strength measures, physical functioning, and self-reported quality of life measurements. Patient-reported treatment-related toxicities will be recorded on a weekly basis.

**Discussion:**

The FiGHTINGF!T trial will provide insight into the potential benefits of a supervised exercise programme in AYAs undergoing cancer treatment. This trial will contribute to the evidence supporting the necessary integration of exercise during cancer treatment, specifically in the under-reported AYA cohort.

**Trial registration:**

This trial was registered retrospectively with the Australia New Zealand Clinical Trial registry (ACTRN12620000663954). Registered on 10 June 2020

**Supplementary Information:**

The online version contains supplementary material available at 10.1186/s13063-021-05616-8.

## Background

On average in Australia, two to three adolescents and young adults (AYAs) aged 15 to 25 years face the news of a cancer diagnosis each day [1]. Between 2010 and 2014, 4843 AYAs were diagnosed with cancer, reported as the leading cause of non-accidental death in this age range [1–3]. While dispute persists internationally regarding the definition of AYAs by age in the cancer context, with ages ranging from 13 to 39 years [4–7], it is widely accepted that this cohort is biologically, physically, and psychosocially unique [5]. Adolescence is a period of transition characterised by significant developmental changes, and as such, a cancer diagnosis at this time can result in unique physical, psychosocial, educational, medical and vocational consequences for the young person which can last well into survivorship [4]. Advances in cancer treatment have led to a rise in overall survival rates for combined AYA cancer diagnoses from 80 to 89% over the last three decades [1]. Despite the obvious benefit borne by these improved survival rates, it also means that a larger proportion of AYA cancer survivors are living for decades with potentially devastating secondary physical and psychosocial consequences of their treatment [8, 9].

AYAs often undergo rigorous treatment protocols resulting in a plethora of physical and psychosocial side effects which can hinder their ability to complete activities of daily living (ADLs). Fatigue, pain, nausea, lack of appetite, vomiting, diarrhoea, loss of strength, impacts on executive function and peripheral neuropathy are commonly cited by AYAs, affecting their ability to function normally [10]. If unresolved or poorly managed, these side effects can contribute to malnutrition, chronic fatigue, cancer-related cachexia and poor physical function, which perpetuates the cycle of inactivity and further deconditioning leading to poor physical functioning and outcomes [11]. Further, this deconditioning can have detrimental effects on AYAs’ engagement in social, vocational, and educational goals during and after cancer treatment [12, 13].

Over the last three decades, evidence supporting the integration of exercise in cancer cohorts has grown exponentially [14–16]. It is now accepted that appropriately tailored exercise is safe and tolerable in a wide range of cancer cohorts, with national and international bodies calling for exercise to be embedded into standard cancer care [15, 17, 18].There is strong evidence that exercise mitigates the known effects of cancer treatments including cancer-related fatigue, muscle wasting, cardiorespiratory decline and psychological distress [19–21]. However, this research is primarily in adult and paediatric cohorts, with a paucity of high-quality evidence available in AYA-specific populations [22]. Several small, single-centre, non-randomised studies and reviews have demonstrated some benefits on the prevention of functional decline, reduction in fatigue and on quality of life variables in AYAs [23–26]. However, while these results are promising, it is recognised that they need to be further explored in a high-quality, randomised controlled trial [22].

In order to address the distinct lack of understanding of integrated exercise on functional capacity and treatment toxicity management in AYAs during cancer treatment, we developed this randomised controlled trial. The primary aim of the study is to determine whether a 10-week individualised exercise intervention is associated with a reduction in the decline of physical fitness (V0_2peak_) experienced by AYAs during cancer treatment, when compared with controls. Secondary aims are to assess the effects of the intervention on muscular strength, body composition, functional capacity, treatment-related toxicities and psychosocial variables. The purpose of this paper is to detail the FiGHTINGF!T study design and protocol based on the Standard Protocol Items for Randomised Trials (SPIRIT) guidelines.

## Methods

### Study design

**Fig. 1:**
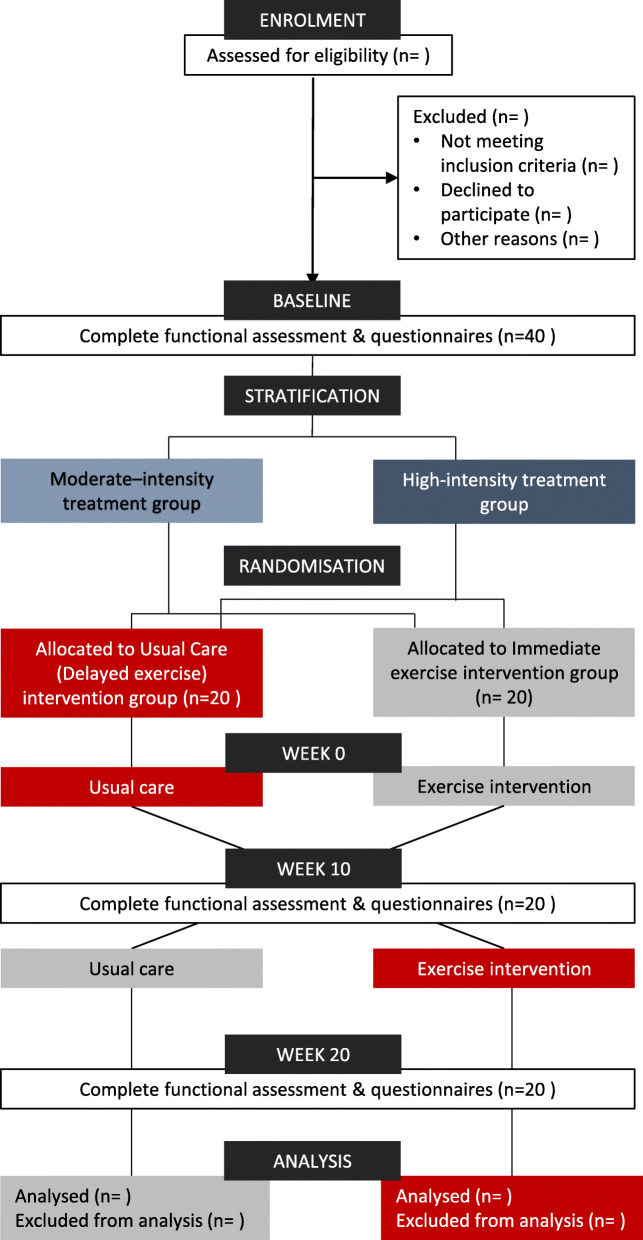
Schematic overview of the FiGHTINGF!T trial

The FiGHTINGF!T trial is a prospective, single-centre, randomised controlled (RCT) cross over, superiority trial. Following baseline assessments, participants will be randomised to either immediate exercise or delayed exercise (usual care) for an initial period of 10 weeks. Following an interim assessment at 10 weeks, participants will cross over to the opposing group and either commence exercise (delayed exercise group) or commence usual care. There will be no wash out period between groups. The study is registered at the Australia New Zealand Clinical Trial Registry (ANZCTR) (ACTRN12620000663954) with participant recruitment for the trial now initiated. Proposed study activities are outlined as per SPIRIT guidelines (Table 1; Additional file 2). The RCT will be conducted over 3 years at the Sir Charles Gairdner Hospital, Perth, Australia. Figure 1 depicts the study design as per the CONSORT guidelines for randomised crossover trials [27].

**Table 1:**
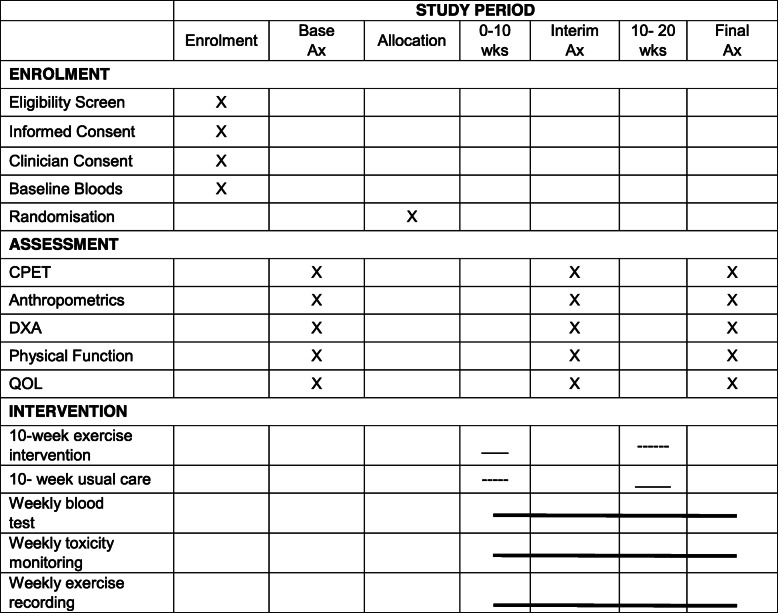
Proposed study activities and summary of assessments

*CPET* Cardiopulmonary Exercise Test; Anthropometrics—height, weight, body mass index, waist and hip circumference; *DXA* dual x-ray absorptiometry; Physical function—1 repetition maximum strength tests, grip strength, 30-s push-ups, 30-s sit ups, 5-repeated sit to stands; *QOL*—EORTC-QLQc30 Hospital Anxiety Depression Score, Pediatric Quality of Life Inventory, International Physical Activity Questionnaire

### Study population

Participants will be recruited through direct referral to the Western Australian Youth Cancer Service (WAYCS) from treating oncology and haematology clinicians based in Perth Metropolitan hospitals. Participants will be deemed eligible if they meet the following criteria:

1. Aged between 15 and 25 years of age as per the Australian definition of AYAs in cancer care
2. Diagnosed with a primary haematological malignancy or solid tumour
3. Scheduled to commence systemic cancer treatment (e.g. chemotherapy, radiation therapy or a combination of both within a ± 2-week period)
4. Medically stable as determined by their treating medical practitioner

Participants will be deemed ineligible to participate if they (1) undergo surgery only, (2) have <6 months life expectancy, (3) have insufficient English competency or a cognitive impairment that would prevent them from participating in the exercise assessments or programmes, (4) are pregnant or lactating and/or (5) have any absolute contraindications to exercise and are deemed inappropriate by their medical treating team.

### Recruitment

This trial will be conducted at Sir Charles Gairdner Hospital. The trial has been approved by the Human Research Ethics Committee and Research Governance Committee at the site (RGS00714) and at the University of Western Australia. All patients who meet the eligibility criteria will be approached within 2 weeks of being diagnosed and will be invited to participate in the study. Potential participants will receive detailed written and verbal information regarding implications of participation, risks and benefits and alternatives to participation. Potential participants will have the opportunity to discuss the study with their treating clinicians and any WAYCS staff prior to undertaking the formal consent process. Those participants who are deemed eligible and agree to participate will be asked to provide written informed consent for study participation. For those participants under 18 years of age, the consent process will be undertaken with their parent/guardian in attendance and consent will be sought from both the participant and their parent/guardian. This process will be undertaken by the Principle Investigator, deemed the most appropriate person to discuss the study and its requirements. Additionally, written medical clearance will be sought from their treating oncologist or haematologist, without which the participant will be deemed ineligible to participate.

### Randomisation and blinding

As this is not a homogenous group, prior to randomisation, participants will be stratified according to intensity of their cancer treatment regimen: high intensity versus low/moderate intensity treatment. Table 2 demonstrates stratification of the most common cancers diagnosed in the AYA age range. Any participant recruited with a diagnosis not listed in Table 2 will be discussed within the research team and treating clinicians to determine the intensity of their treatment regimen. The division between low/moderate versus high intensity relates to the likelihood of low blood counts and degree of immunosuppression from the treatment regimen.

**Table 2:** Stratification strategy of most common adolescent and young adult cancer diagnoses based on treatment regimen

Low/moderate intensity treatment diagnoses	High intensity treatment diagnoses
Hodgkin lymphoma	Acute lymphoblastic leukaemia
Germ cell tumours	Lymphoblastic lymphoma
Gynaecological tumours	Burkitt lymphoma
Non-Hodgkin lymphoma (excluding Burkitt)	Soft tissue and bone sarcoma
Melanoma	Squamous cell carcinoma of the head and neck

Following stratification, participants will be randomised via a computer-generated block randomisation (sealed envelope) procedure. The allocation sequence will be generated and maintained by a member of the research team that is not involved in patient contact or treatment. Both high and moderate intensity treatment groups will be randomised in block sizes of 4 and 8 into the two intervention groups: immediate exercise or usual care (delayed exercise) intervention. The block randomisation will allow for equal groups to be created by the end of the recruitment. Given the nature of the intervention, it is not possible to blind the investigators or the participants to their group allocation. However, the study personnel responsible for conducting primary outcome assessments will be blinded to participant group allocation.

### Intervention

All participants will complete baseline assessments and be randomised prior to commencing (± 2 weeks) systemic treatment. All exercise programmes will be individually prescribed by an Accredited Exercise Physiologist (AEP) and tailored relative to each patient’s presentation. The programmes will follow the American College of Sports Medicine (ACSM) and Exercise and Sports Science Australia (ESSA) guidelines for combined aerobic and resistance exercise for cancer patients [16, 21]. All exercise sessions will be undertaken in a one-on-one basis within a purpose-built gymnasium in the hospital setting and will be supervised by an AEP. No concomitant care or therapies will be specifically permitted (or prohibited) during the trial. However, it is anticipated that participants may exercise outside of the trial, and therefore, all participants will be asked to complete an activity journal to capture any incidental exercise they may perform. The contents of the journal will be checked on a weekly basis by members of the research team. This weekly monitoring will also ensure participants are adhering to the study protocol and associated invention requirements.

#### Immediate exercise group

These participants will commence their exercise programme the week following their baseline assessment and will be required to attend two supervised exercise sessions per week for 10 weeks. Sessions will be approximately 60 min in duration and scheduled at appropriate times relative to participants’ treatment schedules, with exercise conducted prior to or > 24 h following chemotherapy administration. This scheduling strategy is aimed to maintain participant safety, as well as to reduce the burden of additional appointments and limit inconvenience to the participant. Participants will complete a post-intervention assessment at 10 weeks. Following the 10-week interim assessment, participants will then receive no further structured supervised exercise intervention for the remaining 10 weeks, prior to the subsequent final assessment at 20 weeks.

#### Delayed exercise intervention (usual care)

These participants will not be offered any exercise intervention for the initial 10 weeks following baseline assessment. There will be no specific exercise prescription provided to the participants other than that contained in generic information provided by treating teams to all patients as part of the usual standard of care. Participants randomised to this treatment arm will also complete an interim assessment at 10 weeks. Following the interim assessment, participants will then commence bi-weekly supervised exercise sessions for 10 weeks reflecting that offered to the immediate exercise group. Participants will complete final assessments at 20 weeks.

#### Exercise prescription

All exercise sessions will be multi-modal and follow the same format throughout the 10-week intervention. Participants will commence each session with a 5-min warm up, followed by aerobic bouts, a resistance training component and concluding with a 5-min cool down and static stretching period of major muscle groups. The aerobic component of the programme will be based on the participant’s physical capabilities and preference of the modalities available within the gym (including cycling on an upright/recumbent bike, treadmill walking/jogging and/or skipping) and aim to accumulate 20–30 min at moderate intensity over the duration of the session. Intensity will be monitored using heart rate (HR) and rating of perceived exertion (RPE) [28]. Moderate intensity aerobic exercise is considered 60–85% of maximal heart rate (220 − age) [29] or an RPE of 12–14. Participants who are unable to complete 20 min continuously will aim to accumulate the required load in bouts of 5 to 10 min as tolerated. The resistance training component will comprise of 6–8 exercises targeting the major muscle groups of the body. Participants will complete 2–4 sets of 8–15 repetitions, at 60–80% of 1 repetition maximum (1RM) or an RPE of 12–14 [29]. Based on the thorough baseline assessment, any pre-existing injuries or treatment-related effects will be considered when prescribing and progressing all exercises. The sessions will aim to increase in exercise intensity as tolerated. Participants will also be encouraged to engage in aerobic exercise outside of supervised exercise sessions and asked to document their activity in exercise journals to meet exercise recommendations for cancer survivors [16, 21]. Exercise programmes will be modified on a weekly basis as required relative to participants’ treatment schedules and related side effects, as well as changes in their individual physical conditioning throughout the programme. Participants’ vital signs and most recent full blood picture will be reviewed prior to commencing each and every exercise session, to determine their safety for exercise and any need for programme modification as per published guidelines [30]. In the unlikely event any injuries occur during exercise sessions, these will be documented accordingly, and participants will be offered appropriate medical treatment/care without any cost to them.

### Primary outcome measure

The primary outcome of this RCT is cardiorespiratory fitness (V0_2peak_) as measured by a submaximal cardiopulmonary exercise test (CPET). The submaximal test will be conducted in a research setting by trained exercise specialists only, who will be blinded to the participant group allocation [31]. Participants will be instructed to follow their usual diet and incidental activity and abstain from formal exercise in the 24 h prior to testing. Additionally, the research team will review participants’ recent blood test results prior to assessments to ensure results are within the safe parameters for exercise [30]. Prior to commencing the test, participants’ resting heart rate and blood pressure will measured and a heart rate monitor will be attached to the participant (Wahoo Tickr heart rate monitor, Wahoo Fitness LLC, Georgia, USA). Target heart rate (THR) will be calculated using the formula (220 − age) [32] × 0.85 which will be used as criteria to terminate the test unless volitional exhaustion is met prior to this. Participants will be sat on a front access Exertech Ex-10 cycle ergometer (Repco Cycle Company, Huntingdale, Victoria, Australia) and familiarised with the testing protocol. The ramped protocol will have participants commence cycling at 20 watts (W) for 1 min and continue to increase by 20W per minute until they reach their THR or volitional exhaustion. Participants will complete the stage in which they reach their THR unless concluded prior to this. At the end of each minute, heart rate and RPE will be recorded [28]. Participants will complete a 3-min cool down on the bike pedalling with no resistance at a self-selected slow speed. Recovery heart rate and blood pressure will be recorded 10 min after the cessation of the test.

During the test, participants will be required to breathe through a mouthpiece connected to a computerised gas analysis system. The system includes a ventilometer (Universal ventilation meter, Vacumed, Ventura, CA, USA) and oxygen and gas analysers (Ametek Applied Electrochemistry S-3A/1 and CD-3A, AEI Technologies, Pittsburgh, PA) which calculate minute ventilation (V_E_), respiratory exchange ratios (RER) and oxygen and carbon dioxide in expired air at 15-s intervals respectively. Calibration of the equipment will be conducted prior to and following each test using a standard reference gas of known concentration. Values at the conclusion of the test will be recorded in absolute (L/Min) and relative (mL/kg/min) terms. Predicted peak oxygen consumption (V0_2peak_) will be extrapolated using the participants’ heart rate (HR) and V0_2_ from two submaximal stages from the test where a steady state heart rate between 115 and 150 beats per minute is recorded. The slope will be calculated using the ratio of difference between the two submaximal V0_2_ measures and their corresponding heart rates and then be used to extrapolate to predicted maximal heart rate (220 − age) [32–35].

### Secondary outcome measures

#### Strength and physical function

Gross muscular strength will be assessed using a one-repetition maximum test for chest press, decline leg press and seated row, recorded in kilogrammes (kg). Participants with any known bone metastases will be excluded from completing tests that load the location of affected bone [16]. Testing will be conducted by an AEP in accordance with standard ACSM guidelines [29]. Bilateral grip strength will be assessed using a grip strength dynamometer (Jamar Plus Digital, Paterson Medical, IL, USA) with the best of three attempts recorded in kilogrammes. Functional upper limb and lower limb endurance will be assessed using 30-s push-ups and sit ups and a 5-repeated sit to stand (STS) test [29].

#### Anthropometry, body composition and body mass index

Body mass (kg), height (cm), body mass index (BMI, m^2^) and body surface area (m^2^) will be recorded as anthropometric measures. Body mass will be measured to the nearest 0.1kg on electronic scales (Sauter Model EB60, FSE Scientific, Sydney, Australia) with participants asked to remove their shoes and heavy clothing. Body surface area (m^2^) will be obtained from the participants’ chemotherapy charts from within their medical records as it will be used to analyse chemotherapy-related toxicities. Height will be measured using a wall mounted stadiometer to the nearest 0.1cm (Seca 216 Measuring Pole, Birmingham, UK) and BMI will be calculated using recorded body mass and height by dividing body mass (kg) by height in metres squared (m^2^). Waist and hip circumference will be measured using standardised testing protocols [34]. Dual-energy X-ray absorptiometry will be used to record total lean mass (kg) (LM), total fat mass (kg) (FM), proportion of total mass being body fat (%) (%FM) and bone mineral density (BMD) (DXA; Lunar Prodigy, GE Medical Systems, Madison, WI, USA).

#### Patient-reported activity and quality of life (QoL)

Patient-reported physical function, emotional and social wellbeing, fatigue and quality of life will be evaluated using the European Organisation for Research and Treatment of Cancer Quality of Life questionnaire (EORTC QLQ-C30) [36], the Hospital Anxiety and Depression Score (HADS) [37] and an age-specific version of the PEDS-QL and Multidimensional fatigue scale questionnaires [38–40]. Each of the quality of life tools are commonly used and have been validated in AYAs. Participant self-reported physical activity will be assessed using the International Physical Activity Questionnaire [41].

#### Patient-reported treatment-related toxicities

In order to determine that participants meet the safety requirements to complete exercise sessions, they will be required to undergo weekly full blood count (FBC), urea and electrolyte (U&E) and liver function (LFT) blood tests. These results will be reviewed prior to each exercise session to ensure participants meet predetermined safety parameters for exercise [30]. The results will also be reviewed and graded as per the Common Terminology Criteria for Adverse Events (CTCAE Version 5.0) [42] to determine any impact exercise may have on these variables. Further, participants will be contacted weekly by a member of the research team blinded to group allocation to determine if the participant has experienced any additional treatment-related side effects, including but not limited to nausea, vomiting, constipation, pain and fatigue (Additional file 1) [42]. These participant-reported side effects will also be graded by the research team as per the CTCAE version 5.0 [42].

#### Feasibility

The feasibility of the exercise intervention will be determined based on eligibility rate, recruitment rate, compliance to the study protocol, adherence to the exercise intervention and withdrawal rates. Safety will be assessed by monitoring for any adverse or serious adverse events during any of the assessments or supervised exercise sessions. The research team includes a senior and experienced cancer clinician who will provide guidance on individual safety concerns throughout the study. Regular meetings with the research team will be held to review protocol adherence and safety reports. Any adverse events will be reported to the appropriate HREC bodies within an expedient time frame. If in the event of frequent adverse events occurring, which are deemed a result of the intervention, then a collaborative decision can be made to cease the trial. All data will be collected, input and stored in accordance with HREC guidelines as outlined in FiGHTINGF!T Protocol Version 2 03012019. Any modifications to the protocol will be submitted and approved to the relevant HREC bodies and disseminated as appropriate.

### Statistical consideration

A total of 40 participants will be recruited, evaluated and analysed as part of this study. Analysis will be performed using an intention to treat approach [43]. A power analysis using PS Power and sample size calculations Version 3.1.2 has been performed to calculate the sample size required for this study, based on the primary outcome variable VO_2peak_ (ml kg min^−1^). In the absence of comparable published data in AYA patients, similar studies undertaken by Thorsteinsson et al. [44] and West et al. [45] in paediatric and adult patients demonstrated an effect size of 0.80. The standard deviation across multiple studies utilising VO_2peak_ as the primary outcome measure varies from 1.5 ml kg min^−1^ to 5.7 ml kg min^−1^, and therefore, a median value (3.6 ml kg min^−1^) has been utilised for this trial sample size calculation. Assuming a 5% significance level and a power of 0.8, a sample size of 36 patients (18 per group) will be required. An additional 10% has been included (*n*=4) to allow for attrition or unusable data. An interim analysis is planned following 50% of participant recruitment to investigate outcomes between groups.

Both interim and final analysis will employ standard descriptive statistics (M, SD, *n*, %) to summarise data on participant demographics and recruitment rates. Outcome analyses will include Student’s *t*-test, chi-square, correlation, regression and repeated measures ANOVA where relevant to examine differences between groups in the nominated variables, as well as the overall change scores over time. Further statistical analysis will be employed to investigate any associations between exercise-related changes and TRTs. Any clinically relevant covariates will be included in the analyses. All statistical analyses will be conducted using SPSS software (version 20.0, IBM, Armonk, NY, USA) and significance will be set at *p*≤0.05.

### Dissemination

The results for the primary and secondary outcomes of this RCT will be shared regardless of the direction or magnitude of the effect. These outcomes are planned to be published in international high-quality, peer reviewed journals. Results will also be presented at national and international conferences as well as used for teaching purposes to community and university led forums.

## Discussion

To the best of our knowledge this will be the first RCT to examine the benefits of a structured, supervised exercise intervention in AYAs from their cancer diagnosis and throughout their subsequent treatment. Furthermore, it will be the first RCT to explore the potential impact that exercise has on patient-reported treatment-related toxicities. In addition, we will explore any relationship evident between changes in physical functioning and patient-reported treatment-related toxicities.

The design of this study represents both a strength and potential weakness. While a control group is essential to be able to determine any impact that exercise has in the intervention group, the research team deems it unethical to actively encourage sedentary behaviour. In order to address this, the cross over design allows for both groups to be exposed to the exercise intervention and will determine the most appropriate timing for the intervention. Additionally, both groups will be asked to record any physical activity they complete throughout the trial which will be accounted for as potential covariate in the final analysis. Nonetheless, the crossover design will also permit an ability to evaluate any changes in exercise-related beliefs or practices in the immediate exercise group, following their initial 10-week exercise programme.

If it is demonstrated that this exercise intervention prevents functional decline associated with cancer treatment in AYAs, then such programmes can be adopted widely in this cohort. Further, this research will allow clinicians to better understand the potential benefits of exercise on mitigating treatment-related toxicities encouraging targeted research by toxicity, aiming to improve overall treatment tolerance in AYAs.

## Conclusion

There is a paucity of randomised controlled trials investigating exercise interventions in AYA cancer cohorts. The FiGHTINGF!T trial will aim to address the obvious lack of evidence in this area and ultimately provide insight into the potential benefits of exercise in this vulnerable and underreported population.

## Trial status

The FiGHTINGF!T study is currently recruiting. Recruitment commenced in December 2018 and expected to finalise in January 2021. This study is being conducted in accordance with FiGHTINGF!T Protocol Version 2 03012019.

## Supplementary Information

**Additional file 1.** FiGHTINGF!T- Weekly toxicity monitoring example (pdf). Data collection tool for weekly patient-reported toxicity monitoring.

**Additional file 2.** SPIRIT checklist.

**Additional file 3. **FiGHTINGF!T session example.

## Data Availability

Not applicable.

## Abbreviations

1RM: 1 repetition maximum
ACSM: American College of Sports Medicine
ADL: Activity of daily living
AEP: Accredited Exercise Physiologist
ANZCTR: Australia New Zealand clinical trial registry.
AYA: Adolescent and young adult
BMD: Bone mineral density
CPET: Cardiopulmonary exercise tests
CONSORT: Consolidated standards of reporting trials
CTCAE: Common terminology criteria for adverse events
ESSA: Exercise and Sports Science Australia
EORTC QLQ-C30: European Organisation for Research and Treatment of Cancer Quality of Life Questionnaire Core 30
FBC: Full blood count
FM: Fat mass
HADS: Hospital anxiety depression score
HR: Heart rate
HREC: Human Research Ethics Committee
LFT: Liver function tests
LM: Lean mass
M: Mean
PEDS-QL: Paediatric quality of life inventory
QOL: Quality of Life
RCT: Randomised controlled trial
RPE: Rating of perceived exertion
SD: Standard deviation
SPIRIT: Standard Protocol Items: Recommendations for Interventional Trials
THR: Target heart rate
U&E: Urea and electrolyte
VO_2peak_: Peak oxygen uptake
WAYCS: Western Australian Youth Cancer Service
