# Supplementary material for: A supervised exercise intervention during cancer treatment for adolescents and young adults—FiGHTING F!T: study protocol of a randomised controlled trial
Source: Trials. 2021 Oct 3;22:676. doi: 10.1186/s13063-021-05616-8 (PMC8489079; doi:10.1186/s13063-021-05616-8)
Supplement: Supplementary file 1 — Additional file 1. FiGHTINGF!T- Weekly toxicity monitoring example (pdf). Data collection tool for weekly patient-reported toxicity monitoring. [file 13063_2021_5616_MOESM1_ESM.pdf]

FiGHTING F!T Toxicity Classification Sheet.

FiGHTINGF!T

Toxicity Monitoring			
**Phone call completed ☐**	**Weekly blood test ☐**	**Toxicities checked ☐**	**Exercise recorded ☐**
Date:	Date:		

Study ID____________

Week:______________

Common Terminology for Adverse Events Grading (CTCAE v5.0)

Symptom Assessment	Nil	Grade 1	Grade 2	Grade 3	Grade 4	Duration of Sx.
**Fatigue/ Lethargy**		Fatigue relieved by rest	Fatigue not relieved by rest; limiting instrumental ADL's	Fatigue not relieved by rest, limiting self care ADL's.	-	Resolved ☐
**Nausea**		Loss of appetite without alteration in eating habits.	Oral intake decreased without significant weight loss, dehydration or malnutrition.	Inadequate oral caloric or fluid intake, tube feeding, TPN or hospitalisation indicated.	-	Resolved ☐
**Vomiting**		Intervention not indicated	Outpatient IV hydration; medical intervention indicated.	Tube feeding, TPN or hospitalisation indicated.	LTC	Resolved ☐
**Weight loss**		5-<10% from baseline, intervention not indicated.	10-<20% from baseline, nutritional support indicated.	>20% from baseline, tube feeding or TPN indicated.	-	Resolved ☐
**Weight gain**		5-<10% from baseline	10-<20% from baseline	>20% from baseline		Resolved ☐
**Constipation**		Occasional or intermittent symptoms, occasional use of stool softners/laxitives/enema.	Persistent symptoms with regular use of laxatives or enemas, limiting instrumental ADLs	Manual evacuation indicated, limiting self care ADLs	LTC	Resolved ☐
**Diarrhoea**		Increase of <4 stools per day over baseline	Increase of 4-6 stools per day over baseline	Increase of >7 stools per day over baseline; hospitalisation indicated.	LTC	Resolved ☐
**Mucositis**		Asymptomatic or mild symptoms; intervention not indicated.	Moderate pain or ulcer that does not interfere with oral intake	Severe pain; interfering with oral intake	LTC	Resolved ☐
**Peripheral Neuropathy**		Asymptomatic	Moderate symptoms, limiting instrumental ADLs	Severe symptoms, limiting self care ADLs	LTC	Resolved ☐
**Pain**		Mild pain	Moderate pain, limiting instrumental ADLs	Severe pain, limiting self care ADLS	-	Resolved ☐
**Dyspnoea**		Shortness of breath with moderate exertion	Shortness of breath with minimal exertion limiting instrumental ADLs	Shortness of breath at rest limiting self-care ADLs	LTC	Resolved ☐
**Febrile Neutropenia**		-	-	ANC <1.0 with a single temp of >38.3degC or sustained temp >38degC for more than 1 hour.		Resolved ☐
**Mood/ Depression/ Anxiety**		Mild mood changes	Moderate mood changes, limiting instrumental ADLs	Severe mood changes, limiting self-care ADL	LTC; threats of harm to self/ others	Resolved ☐
**Memory/ cognition**		Mild cognitive changes/memory troubles not interfering with work/school/life	Moderate cognitive/memory changes interfering with ADLs but capable of independent living	Severe cognitive/ memory issues, significant impairment of work/work		Resolved ☐
**Insomnia**		Mild difficulty falling asleep, staying asleep or waking up early.	Moderate difficulty falling asleep, staying asleep or waking up early	Severe difficulty falling asleep, staying asleep or waking up early		Resolved ☐
**Other**						Resolved ☐
**Other**						Resolved ☐

Most distressing toxicity this week:____________________________

Common Terminology for Adverse Events Grading (CTCAE 5.0)					
**CTCAE Term**	**Grade 1**	**Grade 2**	**Grade 3**	**Grade 4**	**Duration of Sx**
**Anaemia F (115-160)/ M (135-180) g/L**	Hb <LLN-10g/L<br>**F: 100-114**<br>**M:100-134**	Hb <100-80 g/L<br>**80-99**	<80g/L<br>**Less than 80**	LTC; urgent intervention required	Resolved ☐
**White blood cell decreased (4.0-11.0) x10e9/L**	WCC <LLN-3.0 x10e9/L<br>**3.00-3.99**	<3.0-2.0 x10e9/L<br>**2.00-2.99**	<2.0-1.0 x10e9/L<br>**1.00-1.99**	<1.0 x10e9/L<br>**Less than 1.00**	Resolved ☐
**Platelet count decreased (150-400) x10e9/L**	<LLN-75.0 x10e9/L<br>**75-149**	<75.0-50.0 x10e9/L<br>**50-74**	<50.0-25.0 x10e9/L<br>**25-49**	<25.0 x10e9/L<br>**Less than 25**	Resolved ☐
**Neutrophil count decreased (2.0+) x10e9/L**	<LLN-1.5 x10e9/L<br>**1.50-1.99**	<1.5.0-1.0 x10e9/L<br>**1.00-1.49**	<1.0-0.5 x10e9/L<br>**0.50-0.99**	<0.5 x10e9/L<br>**Less than 0.50**	Resolved ☐
**Lymphocyte count decreased (1.2+) x10e9/L**	<LLN-0.8 x10e9/L<br>**0.8-1.19**	<0.8-0.50 x10e9/L<br>**0.50-0.79**	<0.5-0.2 x10e9/L<br>**0.2-0.49**	<0.20 x10e9/L<br>**Less than 0.20**	Resolved ☐
**Lymphocyte count increased () x10e9/L**	-	>4.0-20 x10e9/L<br>**3.99-20.0**	>20 x10e9/L<br>**>20**	-	Resolved ☐
**Hypernatraemia**	<ULN-150mmol/L	>150-155mmol/L; intervention indicated<br>**151-155/intervention**	>155-160mmol/L; hospitalisation indicated<br>**156-160/hospitalisation**	>160mmol/L; life-threatening consequences<br>**More than 160**	Resolved ☐
**Hyponatraemia mmol/L**	<LLN-130mmol/L<br>**130-134**	125-129mmol/L and asymptomatic<br>**125-129**	125-129mmol/L and symptomatic. 120-124mmol/L regardless of symptoms<br>**125-129 (+symptoms)**<br>**120-124 (+/- symptoms)**	>120mmol/L; life-threatening consequences<br>**Less than 120**	Resolved ☐
**Hyperkalaemia**	<ULN-5.5mmol/L	>5.5-6.0mmol/L; intervention indicated<br>**5.6-6.0/intervention**	>6.0-7.0mmol/L; hospitalisation indicated<br>**6.1-7.0/hospitalisation**	>7.0mmol/L; life-threatening consequences<br>**More than 7.0**	Resolved ☐
**Hypokalaemia**	<LLN-3mmol/L<br>**3.0-3.5**	<3.0-3.5mmol/L; Symptomatic intervention indicated<br>**3.1-3.5/intervention**	<2.5-3.0mmol/L; hospitalisation indicated<br>**2.6-3.0/hospitalisation**	<2.5mmol/L; life-threatening consequences<br>**Less than 2.5**	Resolved ☐
**Blood Bilirubin Increased**	>ULN-1.5xULN<br>**20-30**	>1.5-3.0xULN<br>**31-60**	>3.0-10.0xULN<br>**61-200**	>10. X ULN<br>**>201**	Resolved ☐
**Alanine aminotransferase increased (ALT)**	> ULN -3.0xULN<br>**40 - 120**	>3.0-5.0 x ULN<br>**121-200**	>5.0-20.0 x ULN<br>**201-800**	>20.0 x ULN<br>**>801**	Resolved ☐
**Alkaline phosphatase increased (Alk Phos)**	> ULN -2.5xULN<br>**135-337**	>2.5-5.0 x ULN<br>**338-675**	>5.0-20.0 x ULN<br>**676-2700**	>20.0 x ULN<br>**>2701**	Resolved ☐
**GGT Increased**	> ULN -2.5xULN<br>**60-150**	>2.5-5.0 x ULN<br>**151-300**	>5.0-20.0 x ULN<br>**301-1200**	>20.0 x ULN<br>**>1201**	Resolved ☐
**Hypoalbuminemia**	<LLN-30g/L<br>**30-35**	<20-30g/L<br>**20-29**	<20g/L<br>**Less than 20**	Life-threatening consequences	Resolved ☐
**Hypermagnesaemia**	>ULN-1.23mmol/L<br>**1.10-1.23**	-	>1.23-3.0mmol/L<br>**1.24-3.0**	>3.30mmol/L; life threatening consequences<br>**More than 3.30**	Resolved ☐
**Hypomgnesaemia**	<LLN-0.5mmol/L<br>**0.5-0.7**	<0.4-0.5mmol/L<br>**0.4-0.49**	<0.3-0.4mmol/L<br>**0.3-0.39**	<0.3mmol/L; life threatening consequences.<br>**Less than 0.3**	Resolved ☐
