# Supplementary material for: A supervised exercise intervention during cancer treatment for adolescents and young adults—FiGHTING F!T: study protocol of a randomised controlled trial
Source: Trials. 2021 Oct 3;22:676. doi: 10.1186/s13063-021-05616-8 (PMC8489079; doi:10.1186/s13063-021-05616-8)
Supplement: Supplementary file 3 — Additional file 3. FiGHTINGF!T session example. [file 13063_2021_5616_MOESM3_ESM.docx]

**TRAINING PROGRAM LOG – W1/S1 ­­­­­­­­­­­­­­­ Study No._________ Date: ________**

| **Details** | **Session goals** | | | **Session outcomes** | | | |
| --- | --- | --- | --- | --- | --- | --- | --- |
| **Study No.** | **Total time** | **%HR Max** | **RPE** | **Total mins.** | **RPE** | **Dyspnoea** | **METs** |
| **Date** | 60 mins | 60-80% | 11-14 | 55 minutes | 13 | 5/10 | 5 METs |

| **Cardiovascular exercise (30 mins accumulative):** | | | |  |
| --- | --- | --- | --- | --- |
| **Modality** | **Time (min)** | **HR (bpm)** | **RPE** |  |
| Warm up: Upright stationary bike | 5 mins | 95-110 | 8 |  |
| Aerobic: Treadmill walking at 6km/hr | 20 mins | 110-140 | 12-13 |  |
| Cool down: Upright bike | 5 mins | 110-120 | 10 | **Total: 30** |

| **Resistance Exercises:** Target = 3 sets of 10-15 reps | | | | | | | | | |
| --- | --- | --- | --- | --- | --- | --- | --- | --- | --- |
| 1. | **Leg Press** | 10 reps X | 45kg |  | 10 reps X | 45kg |  | 10 reps X | 45 kg |
| 2. | **Chest- Chest press** | 10 reps X | 4 kg |  | 10 reps X | 4 kg |  | 10 reps X | 4 kg |
| 3. | **Back- Bent over row** | 10 reps X | 5 kg |  | 10 reps X | 5 kg |  | 10 reps X | 5 kg |
| 4. | **Quadriceps X** | reps X | kg |  | reps X | kg |  | reps X | kg |
| 5. | **Shoulder- front raise** | 10 reps X | 2 kg |  | 10 reps X | 2 kg |  | 10 reps X | 2kg |
| 6. | **Hamstrings – Deadlift** | 10 reps X | 15 kg |  | 10 reps X | 15 kg |  | 10 reps X | 15kg |
| 7. | **Biceps – bicep curls** | 10 reps X | 3 kg |  | 10 reps X | 3 kg |  | 10 reps X | 3 kg |
| 8. | **Triceps- cable extension** | 10 reps X | 10 kg |  | 10 reps X | 10kg |  | 10 reps X | 10 kg |
| 9. | **Abdominals – crunches** | 10 reps X | 0 kg |  | 10 reps X | 0 kg |  | 10 reps X | 0 kg |

**TRAINING PROGRAM LOG – W3/S6**

| **Session goals** | | | **Session outcomes** | | | |
| --- | --- | --- | --- | --- | --- | --- |
| **Total time** | **%HR Max** | **RPE** | **Total mins.** | **RPE** | **Dyspnoea** | **METs** |
| 60 mins | 60-80% | 11-14 |  |  |  |  |

| **Cardiovascular exercise (30 mins accumulative):** | | | |
| --- | --- | --- | --- |
| **Modality** | **Time (min)** | **HR (bpm)** | **RPE** |

| Warm up: Upright stationary bike | 5 mins | 95-110 | 8 |  |
| --- | --- | --- | --- | --- |
| Aerobic: Treadmill walking at 6km/hr | 20 mins | 110-140 | 12-13 |  |
| Cool down: Upright bike | 5 mins | 110-120 | 10 | **Total: 30** |

| **Resistance Exercises:** Target = 3 sets of 10-15 reps | | | | | | | | | |
| --- | --- | --- | --- | --- | --- | --- | --- | --- | --- |
| 1. | **Leg Press** | 10 reps X | 45kg |  | 10 reps X | 50kg |  | 10 reps X | 50kg |
| 2. | **Chest- Chest press** | 10 reps X | 4 kg |  | 10 reps X | 5 kg |  | 10 reps X | 5 kg |
| 3. | **Back- Bent over row** | 10 reps X | 5 kg |  | 10 reps X | 6 kg |  | 10 reps X | 6 kg |
| 4. | **Quadriceps X** | reps X | kg |  | reps X | kg |  | reps X | kg |
| 5. | **Shoulder- front raise** | 10 reps X | 2 kg |  | 10 reps X | 2 kg |  | 10 reps X | 2kg |
| 6. | **Hamstrings – Deadlift** | 10 reps X | 20 kg |  | 10 reps X | 20 kg |  | 10 reps X | 20kg |
| 7. | **Biceps – bicep curls** | 10 reps X | 3 kg |  | 10 reps X | 3 kg |  | 10 reps X | 3 kg |
| 8. | **Triceps- cable extension** | 10 reps X | 10 kg |  | 10 reps X | 10kg |  | 10 reps X | 10 kg |
| 9. | **Abdominals – crunches** | 10 reps X | 5 kg |  | 10 reps X | 5 kg |  | 10 reps X | 5 kg |
